# Recombinase polymerase amplification combined with CRISPR/Cas12a technology for rapid on-site detection of duck adenovirus 3

**DOI:** 10.3389/fmicb.2025.1607974

**Published:** 2025-08-06

**Authors:** Qi-Zhang Liang, Wei Chen, Yuhai Bi, Weiwei Wang, Rong-Chang Liu, Qiu-Ling Fu, Guang-Hua Fu, Long-Fei Cheng, Nan-Song Jiang, Ting Zhu, Hong-Mei Chen, Yu Huang

**Affiliations:** ^1^Fujian Academy of Agricultural Sciences/Fujian Provincial Key Laboratory for Avian Diseases Control and Prevention, Institute of Animal Husbandry and Veterinary Medicine, Fuzhou, China; ^2^College of Animal Sciences, Fujian Agriculture and Forestry University, Fuzhou, China; ^3^CAS Key Laboratory of Pathogen Microbiology and Immunology, Institute of Microbiology, Chinese Academy of Sciences, Beijing, China

**Keywords:** DAdV-3, RPA, LFS, CRISPR/Cas12a, on-site detection

## Abstract

Duck adenovirus 3 (DAdV-3) causes liver damage and bleeding, with morbidity rates ranging from 40 to 55% and mortality rates between 35 and 43%. Co-infection with other pathogens complicates disease control, significantly impacting the duck breeding industry. Currently, there have been no effective vaccines or treatments for DAdV-3. Therefore, rapid, specific, and sensitive detection methods are crucial for preventing and controlling this virus. Our study developed a lateral flow strip (LFS) detection method using recombinase polymerase amplification (RPA) and CRISPR/Cas12a. The RPA-CRISPR/Cas12a-LFS method, performed at 37°C, allowed for result visualization without sophisticated equipment. It targeted the DAdV-3 Fiber-2 gene and achieved a detection limit of 3.0 gene copies. Additionally, this method demonstrated high specificity, with no cross-reactivity to eight other avian viruses. The reaction time of RPA-CRISPR/Cas12a-LFS is only 45 min. Analysis of 95 waterfowl samples showed 98.95% consistency and agreement with quantitative polymerase chain reaction using the Fiber-2 RPA-CRISPR/Cas12a-LFS method. These findings highlighted the potential of this user-friendly, rapid, sensitive, and accurate detection method for on-site DAdV-3 detection.

## 1 Introduction

Duck adenovirus 3 (DAdV-3), a non-enveloped double-stranded DNA virus classified within the genus Aviadenovirus of the family Adenoviridae, was first identified in China in 2014 (Zhang et al., 2016). Since its discovery, DAdV-3 has spread widely across Fujian, Zhejiang, Anhui, and Guangdong provinces in China (Shi et al., 2019, 2022; Yin et al., 2022). This virus is known to cause liver necrosis and hemorrhage, with morbidity rates ranging from 40 to 55% and mortality rates between 35 and 43% (Zhang et al., 2016; Shi et al., 2019). Notably, co-infection of DAdV-3 with other pathogens complicates the prevention and control of the disease, severely impacting the duck breeding industry (Yin et al., 2022). Recent research findings have indicated that DAdV-3 is capable of infecting chickens, accompanied by a considerable mortality rate (Zhang Q. et al., 2024; Zhang X. et al., 2024). Despite its widespread prevalence, there are currently no approved vaccines or treatments for DAdV-3 infection. Therefore, timely detection and effective control measures are critical for limiting the spread of DAdV-3 within duck populations and managing the disease effectively.

Compared to traditional pathogen antigen or antibody detection methods, nucleic acid detection technologies are more effective in identifying early stages of disease (Mo et al., 2021; Sciuto et al., 2021). In clinical practice, nucleic acid detection technologies based on clustered regularly interspaced short palindromic repeats (CRISPR) and CRISPR-associated protein 12a (Cas12a) are widely used due to their high specificity for diagnosing infections (Li et al., 2019). In the CRISPR/Cas12a system, CRISPR RNA (crRNA) guides Cas12a to recognize complementary double-stranded DNA sequences containing a protospacer adjacent motif (PAM). Once the target is recognized, Cas12a is activated and cleaves a fluorescent reporter molecule from its quencher, generating a fluorescent signal (Chen et al., 2018). CRISPR/Cas-based nucleic acid detection systems-including DETECTR (Cas12a), SHERLOCK (Cas13a)-achieve exceptional sensitivity and specificity with single-base resolution. These platforms typically integrate with gene amplification methods such as PCR or RPA (Gootenberg et al., 2017; Chen et al., 2018; Mustafa and Makhawi, 2021). Integrating recombinase polymerase amplification (RPA) with CRISPR/Cas12a technology (RPA-CRISPR/Cas12a) significantly enhances the specificity of Cas12a-based detection. This combination reduces the risk of false positives commonly associated with RPA and amplifies the cleavage signal produced by CRISPR/Cas12a (Swarts and Jinek, 2019; Broughton et al., 2020). Furthermore, RPA-CRISPR/Cas12a can be combined with lateral flow strips (LFS) for rapid, sensitive, accurate, and specific on-site visual detection (Yu et al., 2021; Miao et al., 2019).

The genome of DAdV-3 primarily encodes four major structural proteins: Fiber-1, Fiber-2, Hexon, and Penton (Shi et al., 2019). Among these, the two Fiber proteins play crucial roles in DAdV-3 infectivity and pathogenicity (Yin et al., 2019, 2022). Among duck adenoviruses, only DAdV-3 and DAdV-4 possess the Fiber 2 gene. This gene exhibits merely 33% sequence similarity (Huang et al., 2020), rendering it a distinctive genetic marker for differentiating DAdV-3 from other adenovirus types. In this study, various combinations of RPA primers, probes, and crRNA targeting the DAdV-3 Fiber-2 gene were tested for their efficiency. The optimized RPA-CRISPR/Cas12a-LFS method was then evaluated for its sensitivity and specificity. Clinical samples were analyzed using this method, showing 100% concordance with results obtained from conventional quantitative polymerase chain reaction (qPCR). Additionally, this method does not require expensive experimental equipment, making it potentially suitable for clinical field testing. Consequently, it may be valuable for the rapid on-site detection of DAdV-3, thereby aiding in its diagnosis and contributing to the prevention of its spread.

## 2 Materials and methods

### 2.1 Clinical samples, reagents, plasmid and instruments

Ninety five samples of Muscovy ducks (*Cairina moschata*) suspected of DAdV-3 infection were collected in Fujian Province. The sick ducks showed symptoms of depression, a decrease in feed intake and the excretion of yellowish-white loose feces, and their livers were found enlarged and bleeding upon necropsy. Collect the liver of the diseased ducks under aseptic conditions. RNA and DNA extraction was performed using the Animal Total RNA/DNA Isolation Kit from TianLong (Suzhou, China). The LbCas12a protein (a member of the Cas family of proteins and comes from Lachnospiraceae bacteria), EcoRI, and XbaI endonucleases were purchased from New England Biolabs (MA, United States). The RPA kit and LFS was obtained from EZassay Ltd. (Shenzhen, China). The Fiber2 gene was amplified from DAdV-3 genome DNA (strain FJGT01, GenBank No. MH777395.1) and inserted into pcDNA3.1 with a Flag tag. A constant temperature metal bath, purchased from Gingko Biotech (Beijing, China), was set at 37°C for the experiments. Gel imaging was carried out using equipment from Gene Company Limited (MA, United States). DNA and RNA concentrations were measured with the Nanodrop ND-2000 spectrophotometer (NanoDrop Technologies, DE), and fluorescence intensity was measured using the Tecan Infinite M200 plate reader (Männedorf, Sweden).

### 2.2 Design and screening for primers and crRNA of RPA

We used the RPA Design online tool^1^ to design four pairs of primers targeting the DAdV-3 Fiber-2 gene (strain FJGT01, GenBank No. MH777395.1). The primers were selected based on the size of the amplified fragment, which ranged from 120 to 250 base pairs (bp), and primer lengths ranging from 25 to 35 bp. For Cas12a targeting, crRNAs were designed to follow the 3′ end of a PAM sequence (5′-TTTV-3′). Each crRNA sequence (5′-3′) included a T7 promoter (UAAUACGACUCACUAUA), a Cas12a scaffold sequence (UAAUUUCUACUAAGUGUAGAU), and a target sequence of 20–23 bp located after the PAM sequence. We employed the CrRNA Design online tool^2^ to design crRNAs and choose the optimal one according to prediction scores. Primers and crRNAs were synthesized by EZassay Ltd. (Shenzhen, China). Detailed sequence information used in this study is summarized in Table 1.

**Table 1:** The sequence of RPA primers and crRNA.

Name	Sequence (5′-3′)	Position
DAdV-3-Fibir2-RPA-F1	CACTTACAATCGACCCTGGCAAAGGACTGGA	605–635
DAdV-3-Fibir2-RPA-R1	GTCTTCTGAAGTGTGTCAGATCCACTCGTAA	824–854
DAdV-3-Fibir2-RPA-R2	GAAGTGTGTCAGATCCACTCGTAAAGACATA	817–847
DAdV-3-Fibir2-RPA-F2	ATCGATCAATCACTCTCCGTTAGAACTAATC	643–673
DAdV-3-Fibir2-RPA-R3	CACTTGGGCTTGTGTCTTCTGAAGTGTGTCA	837–867
DAdV-3-Fibir2-RPA-R4	CCCTGCTCCGCAGCACACTTGGGCTTGTGTC	852–882
DAdV-3-Fibir2-crRNA	UAAUUUCUACUAAGUGUAGAUUCUCCUUGAGGAUUAGUUCUAAC	661–683

### 2.3 RPA reactions

The RPA reaction was executed utilizing the RPA Kit in accordance with the protocol provided by the manufacturer. In summary, the reactions were performed in a total volume of 20 μL, comprising 10.0 μL of reaction buffer, 6.0 μL of RNase/DNase-free water (ddH_2_O), and 0.5 μL each of upstream and downstream primers at a concentration of 20 μM/L. The RPA reaction mixture was then incubated at 37°C in a metal bath for a duration of 20 min.

### 2.4 Cas12a detection reactions

The reactions were performed with 5 μL of RPA products, 1 μL of LbCas12a protein, 2 μL of cleavage buffer, 1 μL of crRNA, 0.6 μL of single-stranded DNA (ssDNA) reporter (4 μM), and ddH_2_O to a total volume of 20 μL. The reaction mixture was incubated in a metal bath at the temperature of 37°C for 25 min. The Black Hole Quencher (BHQ)-labeled ssDNA probe (FAM-TTATT-BHQ) and the biotin-labeled ssDNA probe (FAM-TTATT-Biotin) were synthesized and purified by EZassay Ltd. (Shenzhen, China). The activity of Cas12a DNA endonuclease was evaluated by employing blue light of 450 nm in wavelength, along with fluorescence signals where the excitation wavelength was 492 nm and the emission wavelength was 521 nm, respectively.

### 2.5 Lateral flow detection

After completing the amplification and Cas12a detection reactions, 2 μL of the reaction product was mixed with 78 μL of dilution buffer. The test results were observed 2 min after adding a lateral flow test strip to the reaction mixture. DNA was detected using the elimination method. A single band appearing on the control line (C) indicated a positive result, while bands appearing on both the control line (C) and the test line (T) indicated a negative result.

### 2.6 Optimization of CRISPR/Cas12a detection reactions

To optimize the reaction concentrations of Cas12a and crRNA, the CRISPR/Cas12a reactions were conducted in different concentrations of Cas12a (25, 50, 100, 150, and 200 nmol/L) and crRNA (25, 50, and 100 nmol/L), and each reaction products was tested by reading the fuorescence signal to determine the optimal concentration.

### 2.7 Sensitivity and specificity of RPA-CRISPR/Cas12a detection reactions

To investigate the sensitivity of the RPA-CRISPR/Cas12a reaction, 10-fold serial dilutions of the pcDNA3.1-DAdV-3 Fiber-2-Flag plasmid standard were used as templates for the RPA reaction, whereas ddH2O was used as a negative control. The nucleic acids extracted from multiple viruses that seriously threaten poultry health, namely, Muscovy duck parvovirus (MDPV), Duck circovirus (DuCV), Adenovirus type 4 (AdV-4), Duck tembusu virus (DTMUV), Egg drop syndrome virus (EDSV), Duck hepatitis virus (DHV), Duck astrovirus (DAstV), and Duck plague virus (DPV), were detected to evaluate the analytical specificity of the RPA-CRISPR/Cas12a assay.

### 2.8 Statistical analysis

GraphPad Prism 9 software (GraphPad Software, Inc.) was utilized to analyze the data. Statistical significance was evaluated through the application of two-tailed t-tests. The results were shown as mean ± standard error of the mean (SEM) based on three independent experiments, with *p* values lower than 0.05 being considered significant.

## 3 Results

### 3.1 Mechanism of the elimination method for strip detection

**Figure 1:**
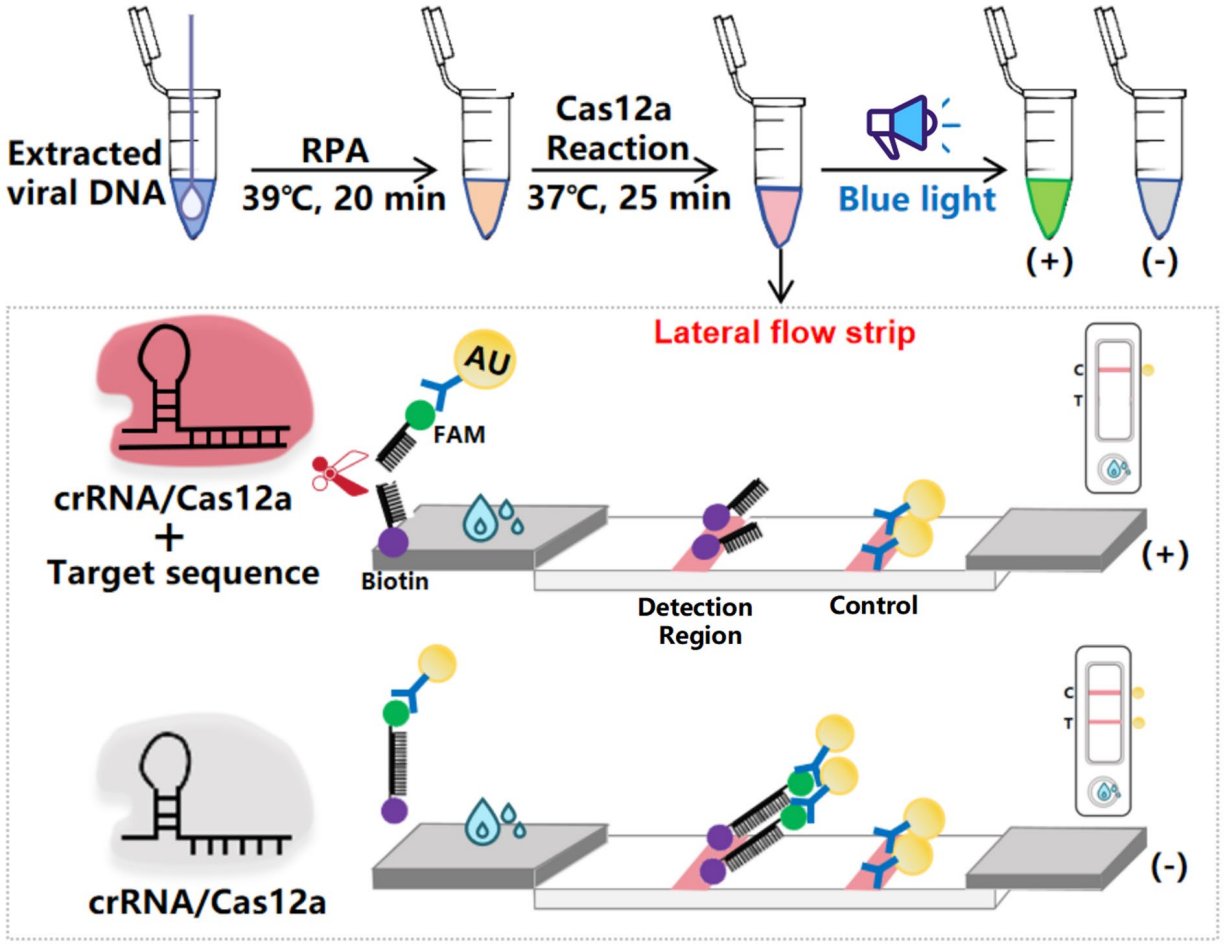
Schematic diagram of the elimination method for LFS detection. The ssDNA probe, labeled with 5′-gold-Ab-FAM and 3′-biotin, was used for LFS detection, with the top half representing the inactivated state and the bottom half representing the activated state.

Upon completion of the reaction, the CRISPR/Cas12a solution was introduced to the sample pad of the test strip. The principle of the strip detection method for DAdV-3 DNA is illustrated in Figure 1. In this method, gold nanoparticles conjugated with FAM antibodies bind to the ssDNA probe on the test strip, forming a detection probe designated as 5′-gold-Ab-FAM-ssDNA-biotin-3′. Streptavidin on the control (C) line captured the biotin-labeled probes, resulting in the formation of a visible gold line. In the presence of DAdV-3 genes in the sample, the FAM-labeled gold particles and biotin moieties were cleaved from the ssDNA probe due to Cas12a-mediated cleavage of the reporter RNA. Consequently, the FAM-labeled gold particles did not remain on the test (T) line, leading to the absence of detectable bands, which indicates a positive result. In the absence of DAdV-3 genes, the FAM-conjugated gold particles and biotin-labeled probes remained intact, resulting in bands on both the C and T lines, indicating a negative result. Conversely, the absence of bands on the C line rendered the test strip invalid.

### 3.2 RPA primer screening

**Figure 2:**
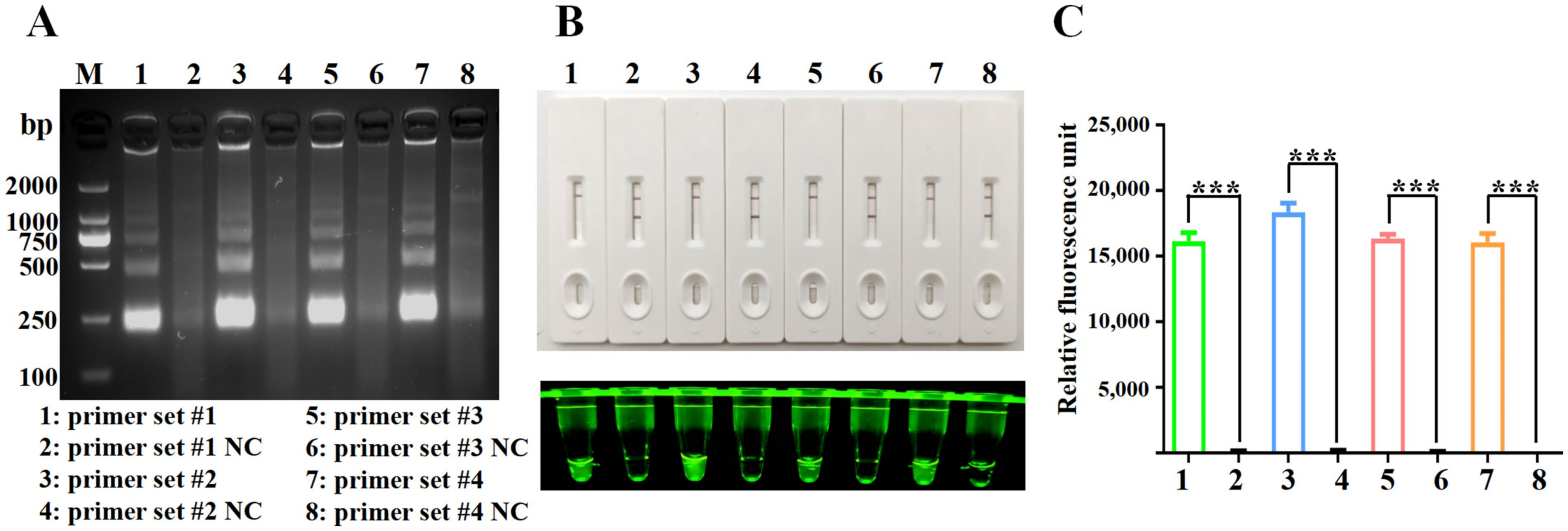
Screening of RPA primers. **(A)** RPA products were verified by 1% agarose gel electrophoresis. M: DL2000 DNA marker; Lane 1: primer set #1; Lane 3: primer set #2; Lane 5: primer set #3; Lane 7: primer set #4. Lanes 2, 4, 6, and 8 are negative controls with water as the template. **(B)** CRISPR/Cas12a detection using the four primer sets. The top panel shows LFS detection, while the bottom panel displays fluorescence intensity. **(C)** Fluorescence values for CRISPR/Cas12a detection using the four primer sets. Error bars indicate SEM (two-tailed t-test, ***p < 0.001; n ≥3).

The evaluation of oligonucleotide sequences is essential for RPA, especially for assessing primer efficacy. Four primer pairs targeting the Fiber-2 gene were initially tested, and their amplification products were separated using agarose gel electrophoresis. Figure 2A shows the successful amplification of a DNA fragment of the expected size (250 bp) with all primer sets. The Cas12a-LFS detection results (Figure 2B, upper) indicate that all tests with the four primer pairs (lines 1, 3, 5, and 7) yielded positive results, whereas the no-template control showed negative results. Subsequent Cas12a fluorescence analysis (below part of Figures 2B,C) revealed that primer set #2 exhibited the highest fluorescence intensity and values among the evaluated sets. Therefore, primer set #2 was selected for further experimental investigations.

### 3.3 Optimization of Cas12a and crRNA concentrations

**Figure 3:**
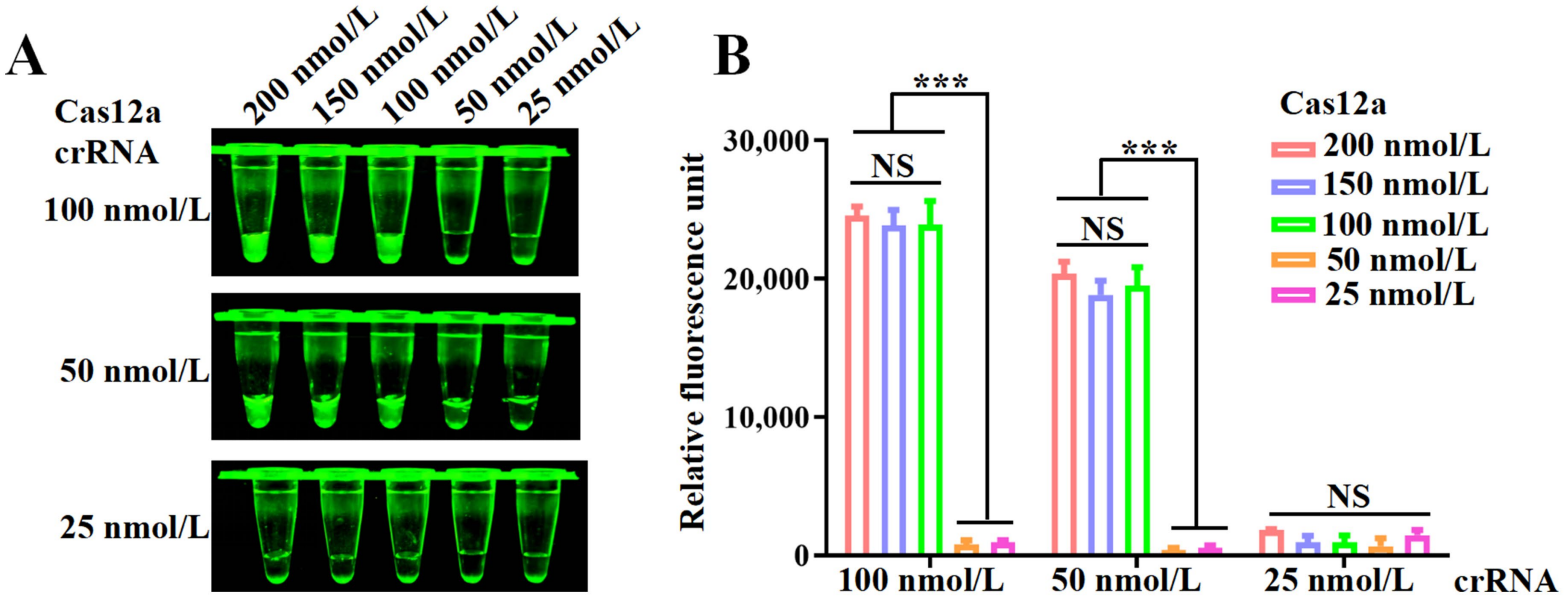
Optimization of the RPA and CRISPR/Cas12a system. **(A)** Fluorescence intensity of CRISPR/Cas12a reactions mediated by different concentrations of Cas12a and crRNA. **(B)** Fluorescence values measured using a fluorescence microplate reader. Error bars indicate SEM (two-tailed t-test, ***p < 0.001; *n* ≥3).

To optimize the concentrations of Cas12a and crRNA in the CRISPR/Cas12a system, a series of reactions were conducted using varying concentrations of Cas12a (25, 50, 100, 150, and 200 nmol/L) and crRNA (25, 50, and 100 nmol/L). As shown in Figure 3A, the reaction group with 100 nmol/L Cas12a exhibited higher fluorescence intensity compared to the group with 50 nmol/L Cas12a. Furthermore, under the same conditions, the group with 50 nmol/L crRNA showed stronger fluorescence intensity than the group with 25 nmol/L crRNA. Despite these observations, the fluorescence values presented in Figure 3B did not reveal statistically significant differences with increasing concentrations of Cas12a and crRNA. Therefore, for subsequent experiments, concentrations of 100 nmol/L for Cas12a and 50 nmol/L for crRNA were selected based on these results.

### 3.4 Sensitivity of RPA-CRISPR/Cas12a method

**Figure 4:**
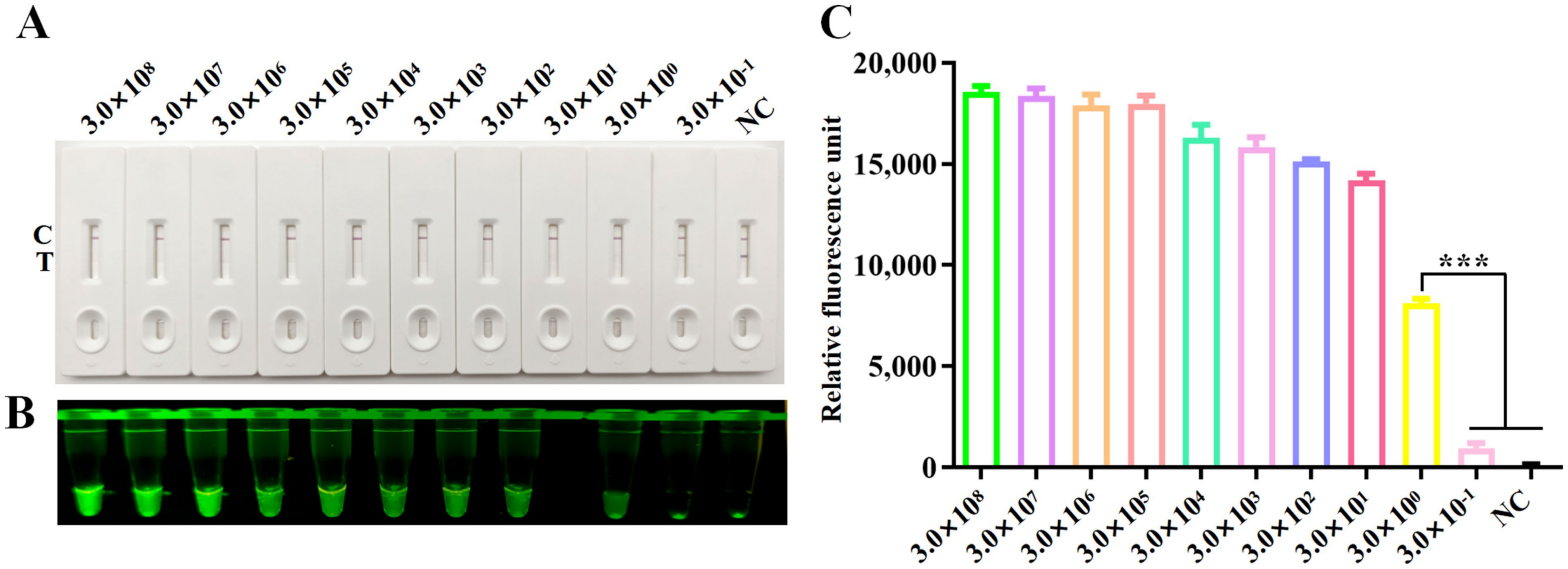
Sensitivity analysis. Sensitivity of CRISPR/Cas12a reaction for detecting the Fiber-2 gene across gradient concentrations ranging from 3.0 × 10^8^ copies/μL to 3.0 × 10^−1^ copies/μL. NC denotes negative control. Sensitivity of RPA-CRISPR/Cas12a LFS detection **(A)** was verified by blue light detection **(B)** and fluorescence detection **(C)**. Error bars indicate SEM (two-tailed t-test, ***p < 0.001; *n* ≥3).

The analytical sensitivity of the CRISPR/Cas12a system was assessed using the plasmid pcDNA3.1-DAdV-3 Fiber-2-Flag constructed in our laboratory as templates, diluted from 3.0 × 10^8^ to 3.0 × 10^−1^ copies/μL. RPA amplification was done on each dilution, followed by CRISPR/Cas12a-LFS detection. The detection limit of the CRISPR/Cas12a method was established at 3.0 × 10^0^ copies/μL, as indicated by the complete absence of the test line on the test strip (Figure 4A) and significant differences in fluorescence intensity (Figure 4B) and fluorescence values (Figure 4C) between the 3.0 × 10^0^ copies/μL sample and the 3.0 × 10^−1^ copies/μL sample.

### 3.5 Specificity of RPA-CRISPR/Cas12a detection method

**Figure 5:**
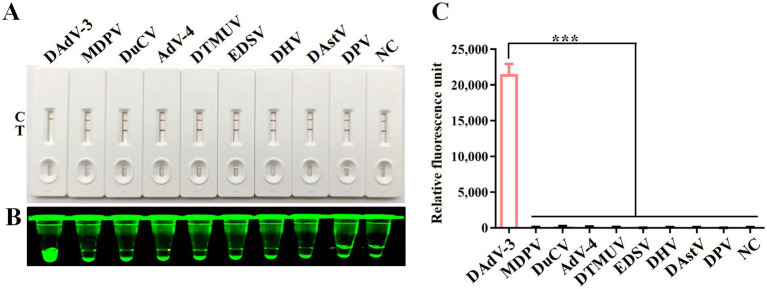
Specificity analysis. DNA or RNA from MDPV, DuCV, AdV-4, DTMUV, EDSV, DHV, DAstV and DPV was used as templates for RPA-CRISPR/Cas12a reaction. Specificity of LFS detection **(A)** was verified by blue light detection **(B)** and fluorescence detection **(C)**. Error bars indicate SEM (two-tailed t-test, ***p < 0.001; *n* ≥3).

To validate the specificity of the CRISPR/Cas12a detection method, nucleic acid samples from eight duck viruses other than DAdV-3 were tested, including MDPV, DuCV, AdV-4, DTMUV, EDSV, DHV, DAstV and DPV. The results from the CRISPR/Cas12a reaction using LFS (Figure 5A), blue light detection (Figure 5B), and fluorescence detection (Figure 5C) consistently showed that only DAdV-3 viral DNA produced a positive signal, confirming the reliability and specificity of the method.

### 3.6 RPA-CRISPR/Cas12a-LFS detection of clinical samples

**Figure 6:**
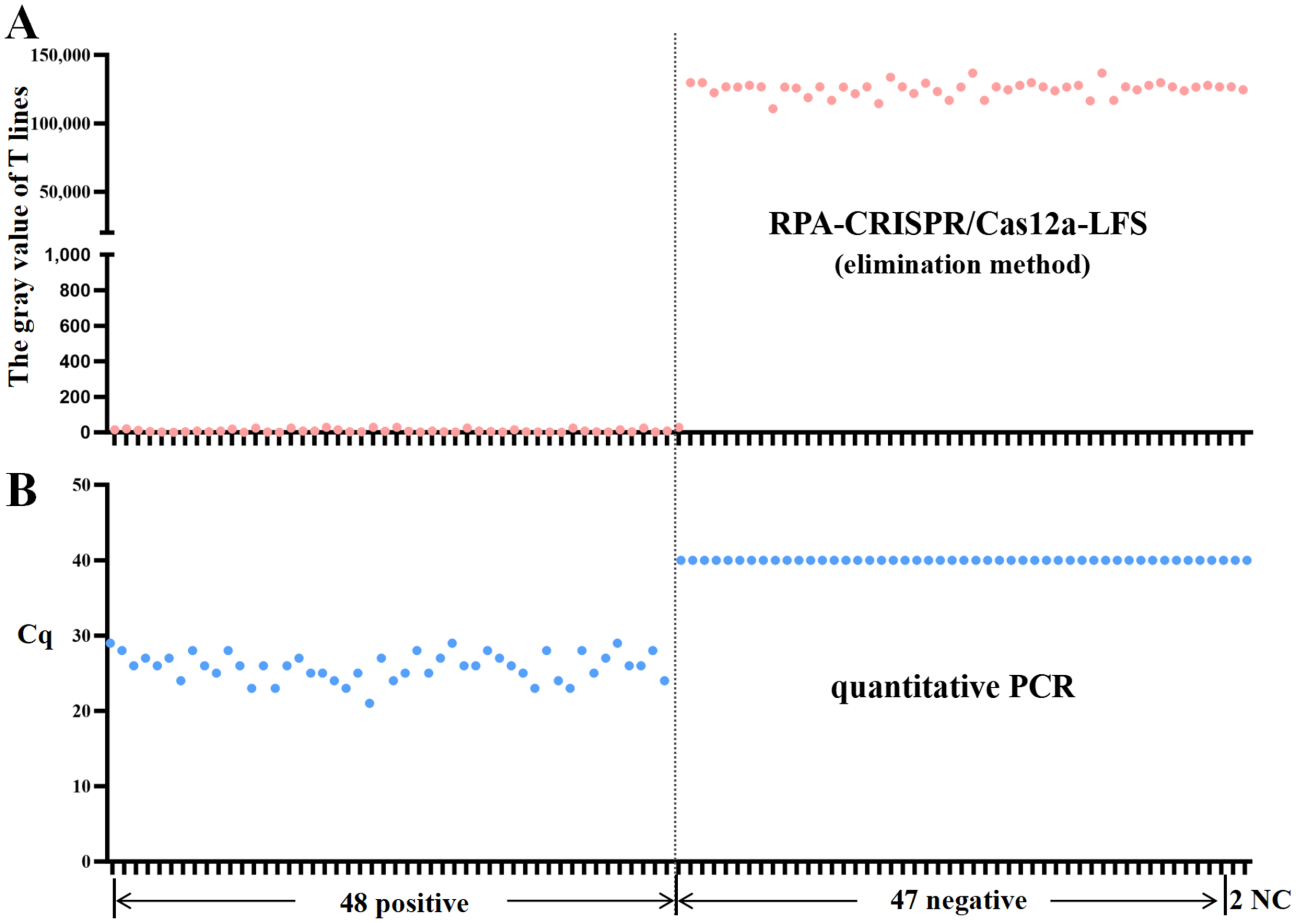
RPA-CRISPR/Cas12a-LFS detection of clinical samples. The gray value of T lines on LFS were measured by Image J software. The presence of a grayvalue indicates the existence of a band, which means the detection result is positive. Results of RPA-CRISPR/Cas12a-LFS detection **(A)** and qPCRdetection **(B)** of 95 clinical samples are shown above. NC indicates negative control.

To evaluate the performance of the RPA-CRISPR assay for detecting DAdV-3 in clinical samples, 95 duck samples were analyzed. The detection principle of the test strips used in this study is as described in Figure 1. The grayscale values of the T lines on lateral flow strips (LFS) were quantified using ImageJ software (National Institutes of Health, Bethesda, MD, United States). The detection of a grayscale value signifies the presence of a band, thereby indicating a positive detection result. The results showed that 49 samples were strongly positive and 46 samples were negative (Figure 6). In order to verify the reliability of the test strip for detecting DAdV-3, the qPCR method (Wan et al., 2018) developed by our team was used for verification detection. The results demonstrate that the concordance rate between the two methods can attain an impressive 98.95% (Table 2). These findings confirm the reliability of the RPA-CRISPR/Cas12a-LFS method for detecting DAdV-3 in clinical samples.

**Table 2:** The performance of RPA-CRISPR compared with qPCR.

Detection method		qPCR			CR
		Positive	Negative	Total	
RPA-CRISPR/Cas12a-LFS	Positive	48	1	49	98.95%
	Negative	0	46	46	
	Total	48	47	95	

## 4 Discussion

The control and management of DAdV-3 critically depend on the early detection of the virus in infected ducks, especially given the lack of effective vaccines and treatments. Current detection techniques, including PCR, qPCR, and loop-mediated isothermal amplification (LAMP) (Sciuto et al., 2021 Chen et al., 2022, 2024; Wang et al., 2024), are commonly used for identifying DAdV-3. However, PCR and qPCR methods are labor-intensive, require costly equipment, and often necessitate specialized personnel for accurate interpretation of results. LAMP assays have garnered considerable attention due to their enhanced sensitivity for detecting DAdV-3. However, they are challenged by complex primer design, limited reaction temperature ranges, and the potential for false-positive results. Therefore, there is an urgent need for the development of a rapid, accurate and simple diagnostic test for identifying DAdV-3 infections.

In recent years, RPA has emerged as a novel isothermal nucleic acid amplification technique, utilizing key components such as recombinase, DNA polymerase, and single-stranded DNA binding protein. RPA offers a significant advantage in its increased tolerance to PCR inhibitors, making it a preferred platform for nucleic acid amplification. Additionally, the rapid and straightforward nature of the RPA assay accelerates the reporting process. The detection speed of mere RPA amplification is relatively rapid. Nevertheless, its coincidence rate with other methods is rather low, and there is also a deficiency in the accuracy of detection (Liu et al., 2023) The CRISPR-Cas-based assay, known for its high specificity in gene editing, is widely used for infection diagnosis. The SHERLOCK platform developed by Feng Zhang’s group pioneered CRISPR-based molecular diagnostics through Cas13-RPA integration, demonstrating high sensitivity for RNA virus detection in field settings. Similarly, Jennifer Doudna’s team created the DETECTR system leveraging Cas12a’s trans-cleavage activity coupled with RPA, which extended this approach to DNA target quantification with single-base specificity for applications like HPV genotyping (Gootenberg et al., 2017; Chen et al., 2018; Mustafa and Makhawi, 2021).

Detection of CRISPR-Cas activity can be achieved through fluorescence readers, LFSs, or lateral flow-based systems (Swarts and Jinek, 2019; Broughton et al., 2020). In the field of waterfowl and poultry research, the CRISPR-Cas-based system has been documented solely for the detection of avian influenza virus (AIV), duck hepatitis A virus 3 (DHAV-3), and novel duck reovirus (NDRV) (Zhou et al., 2023; Chen et al., 2022; Zhang Q. et al., 2024; Zhang X. et al., 2024). To the best of our knowledge, there have been no reports to date on the application of this system for the detection of duck adenovirus 3 (DAdV-3).

In this study, CRISPR/Cas12a was targeted at the highly conserved DAdV-3 Fiber-2 gene, where multiple PAMs were identified. Cas12a reactions were efficiently mediated by crRNA designed based on these PAM sequences. We also optimized various Cas12a reaction parameters, including primer sequences and Cas12a/crRNA ratios, allowing for successful visualization under blue light and on a LFS. In this RPA-Cas12a system, as few as three copies of the target could be detected. Notably, the entire process—from nucleic acid extraction to RPA reaction to CRISPR/Cas12a reaction to final LFS results—can be completed within 1 h. Additionally, the system eliminates the need for expensive equipment and specialized technicians, as results can be observed with the naked eye. To evaluate detection specificity, eight different types of duck viruses were included in the assessments. The RPA-CRISPR/Cas12a method showed no cross-reactivity with these other duck viruses, underscoring its high specificity and significant potential for clinical detection. To further validate the reliability of this approach, 95 clinical samples were tested using both the RPA-CRISPR/Cas12a-LFS method and conventional qPCR. The results demonstrated a 98.95% concordance rate between the two techniques.

In the future, as the biomanufacturing industry advances and production costs for primers and reagents decrease, this technology will inevitably see wider application. For on-site testing, freeze-dried powder can be used to maintain reagent stability and result reproducibility. Concurrently, improved expertise among animal husbandry and veterinary practitioners will enhance reproducibility of laboratory findings.

## Data Availability

The original contributions presented in the study are included in the article/supplementary material, further inquiries can be directed to the corresponding authors.

## Footnotes

^1^ https://www.ezassay.com/primer

^2^ https://www.ezassay.com/rna
